# Significance of Peripheral Perfusion Changes During Remote Ischemic Conditioning in Critically Ill Patients

**DOI:** 10.3390/jcm15041624

**Published:** 2026-02-20

**Authors:** Mantas Jaras, Edvinas Chaleckas, Zivile Pranskuniene, Tomas Tamosuitis, Andrius Pranskunas

**Affiliations:** 1 Department of Intensive Care Medicine, Lithuanian University of Health Sciences, 50161 Kaunas, Lithuania; 2 Health Telematics Science Institute, Kaunas University of Technology, 51423 Kaunas, Lithuania; 3 Institute of Pharmaceutical Technologies, Lithuanian University of Health Sciences, 50166 Kaunas, Lithuania; 4 Department of Drug Technology and Social Pharmacy, Lithuanian University of Health Sciences, 50166 Kaunas, Lithuania

**Keywords:** remote ischemic conditioning, perfusion index, endothelial function, fluid responsiveness, blood pressure cuff deflation

## Abstract

**Objectives**: This study aims to evaluate whether changes in perfusion index (PI) after the first deflation of the blood pressure cuff during remote ischemic conditioning (RIC) are associated with passive leg raising (PLR)-induced changes in stroke volume. In addition, we compared PI changes after cuff deflation during RIC between critically ill patients and healthy controls. **Methods**: This prospective, single-center study was conducted in a mixed ICU at a tertiary teaching hospital. Patients aged >18 years admitted to the ICU, monitored using calibrated pulse contour analysis, and scheduled for a PLR test as decided by the attending physicians were included. The PI was measured after blood pressure cuff deflations during RIC (3 cycles of brachial cuff inflation to 200 mmHg for 5 min, followed by instantaneous deflation to 0 mmHg for another 5 min) in the supine position after PLR. Preload responsiveness was defined as a ≥10% increase in the stroke volume index (SVI) during PLR. Data were compared with a healthy control group. **Results**: Thirty-three patients were included (median age 62; 45% in shock; 55% mechanically ventilated). When comparing critically ill patients with healthy volunteers, the maximum PI change (dPImax) and the time to reach it were higher in critically ill patients after the first and second cuff deflations (*p* < 0.05). However, after the third deflation, the difference was no longer significant. Following the first deflation, dPImax was significantly correlated with SVI changes during PLR (r = 0.63, *p* < 0.001). After the cuff was first deflated, we detected a PI cutoff with a positive SVI response (≥10%) during PLR, with a sensitivity of 64% and a specificity of 94% (area under the receiver operating characteristic curve 0.752; 95% CI, 0.564–0.940; *p* = 0.008). **Conclusions**: The maximum change in perfusion index following brachial blood pressure cuff deflation after five minutes of inflation may serve as a promising noninvasive bedside indicator of preload responsiveness in critically ill patients. Additionally, the observed normalization of PI kinetics during RIC suggests possible acute modulation of vascular reactivity, though further research is needed to confirm an association between PI changes and endothelial function.

## 1. Introduction

Remote ischemic conditioning (RIC) is a non-invasive technique in which brief, controlled episodes of ischemia and reperfusion are applied to a limb to increase the resistance of a distant organ (such as the heart, brain, or kidney) to ischemia-reperfusion injury. Originally developed for myocardial ischemia–reperfusion injury [1], RIC is now being studied in various clinical settings, such as major surgery, stroke, and critical illness, due to its good safety profile and low resource needs [2,3]. The most common method of RIC is 3 to 4 cycles of inflation for 5 min, followed by deflation for 5 min, using a standard blood pressure cuff on the upper arm or thigh. Although many mechanistic pathways have been suggested, including neural reflexes, circulating humoral factors, modulation of endothelial function, and reduction of systemic inflammation, the practical use of these diverse effects as bedside monitoring tools remains incomplete [4,5,6]. There is a pressing need for a simple, real-time, noninvasive indicator that can assess the microcirculatory and vasoregulatory effects of RIC in both patients and healthy individuals.

The perfusion index (PI), defined as the ratio of pulsatile (AC wave, arteries) to non-pulsatile (DC wave, capillaries, venous vessels, bone and soft tissues) portions of pulse wave derived from the photoplethysmographic data collected by pulse oximeters, provides a continuous, operator-independent measure of peripheral blood flow, blood volume, and vascular tone [7,8,9]. PI indicates the local blood volume change during systole and varies with systemic and local hemodynamic conditions [9]. PI results from the local perfusion balance between peripheral factors, mainly vascular tone, and central factors, such as stroke volume.

Zahedi and colleagues [10] demonstrated that photoplethysmographic wave analysis can detect flow-mediated dilation (FMD) response during brachial cuff deflation after 4 min of inflation in the peripheral circulation, similar to measuring the brachial artery diameter with ultrasound. The proposed technique using a finger photoplethysmogram can be employed for rapid, non-invasive assessment of endothelial function as a low-cost, less operator-dependent alternative to ultrasound.

Evidence suggests that RIC can acutely affect vasoreactivity and endothelial function, indicating that PI may increase after a standard RIC stimulus through decreased vasoconstriction and improved microvascular conductance [11,12]. However, the extent, timing, and durability of PI responses to RIC, and whether these responses differ between healthy and critically ill patients, are not well understood.

Beyond traditional methods of assessing endothelial function—which often rely on complex measurements of vascular diameter—there is a clinical mandate for a simple, bedside-ready approach to determine fluid tolerance. While fluid resuscitation aims to optimize cardiac output, its ultimate goal is to restore tissue perfusion. Current dynamic preload assessments in the ICU face significant hurdles: invasive monitors (e.g., PiCCO_2_) are costly and carry procedural risks, while non-invasive alternatives such as bedside echocardiography are highly operator-dependent [13]. Furthermore, the ‘gold standard’ Passive Leg Raising (PLR) test is often unfeasible in patients with spinal instability or limb trauma. Consequently, the combination of a standard blood pressure cuff and pulse oximetry-derived PI may represent a promising, non-invasive, inexpensive alternative for assessing fluid deficits, vascular reactivity, and the efficacy of RIC.

Blood pressure cuff deflation during the RIC procedure causes temporary vasodilatory shifts that can reveal how responsive the local vasculature is to changes in preload and what volume reserve it has in the limb. Possibly, if PI increases at the necessary level during blood cuff deflation, it suggests that the vasculature can recruit additional perfusion with improved hemodynamics—an indicator aligned with fluid responsiveness. Conversely, a muted PI response may indicate limited cardiovascular reserve and a reduced likelihood that a fluid bolus will improve cardiac output. Previous studies have shown that PI changes can correlate with changes in cardiac output during various maneuvers, such as PLR [14].

Therefore, this study aims to evaluate whether changes in PI after the first deflation of the blood pressure cuff during RIC are associated with PLR-induced changes in stroke volume. In addition, we compared PI changes after cuff deflation during RIC between critically ill patients and healthy controls.

## 2. Materials and Methods

### 2.1. Participants

This single-center prospective study was conducted in an 18-bed mixed ICU at a tertiary teaching hospital (The Hospital of the Lithuanian University of Health Sciences). The study was approved by the Kaunas Regional Biomedical Research Ethics Committee (No. BE-2-98) and conducted in accordance with the Declaration of Helsinki. Written informed consent was obtained from the patients or their next of kin, consistent with applicable laws.

Patients were included in the study if they met the following criteria: aged over 18 years, admitted to the ICU, monitored with a transpulmonary thermodilution device with calibrated pulse contour analysis (PiCCO_2_, Getinge AB, Gothenburg, Sweden), and scheduled for a PLR test as decided by the attending physician. The exclusion criteria were pregnancy, advanced malignancy, peripheral artery disease affecting both arms, head trauma, deep vein thrombosis in the lower limbs, and intra-abdominal hypertension, defined as an intra-abdominal pressure (IAP) greater than 12 mmHg. Individuals considered inappropriate for enrollment due to other factors, such as bleeding, escalating vasopressor doses, an urgent need for a fluid bolus, and an absent or unstable plethysmographic signal, were also excluded.

For the healthy control group, we recruited 14 healthy, recreationally active volunteers. Individuals with an acute illness, known cardiovascular disease, or risk factors for thromboembolism were excluded. Participants were advised to avoid caffeine, alcohol, and tobacco for 12 h before the experiment.

### 2.2. Study Protocol

The participants were placed in a semirecumbent position for at least 2 min with the backrest of the bed folded to an angle of 45°. Systemic hemodynamics, including the stroke volume index (SVI) and heart rate (HR), were assessed. The SVI in critically ill patients was measured via transpulmonary thermodilution with a PiCCO_2_ monitor (Getinge AB, Gothenburg, Sweden). The pressure sensors connected to the arterial and central venous catheters were positioned on the patient’s upper arm at the estimated level of the right atrium. During PLR the SVI was measured via pulse contour analysis. In healthy volunteers, hemodynamic parameters were assessed using an ICON monitor (Osypka Medical GmbH, Berlin, Germany), which employs the Electrical Cardiometry method to estimate stroke volume and cardiac output through noninvasive, continuous measurements of thoracic bioimpedance [15].

The body was then moved to a supine position with subsequent passive positioning of both legs raised 45° from the bed for 2 min. The highest SVI values during the PLR test were recorded. Preload responders and nonresponders were defined as those with SVI increases of ≥10% and <10%, respectively. The patient was subsequently repositioned to a semirecumbent position.

**Figure 1:**
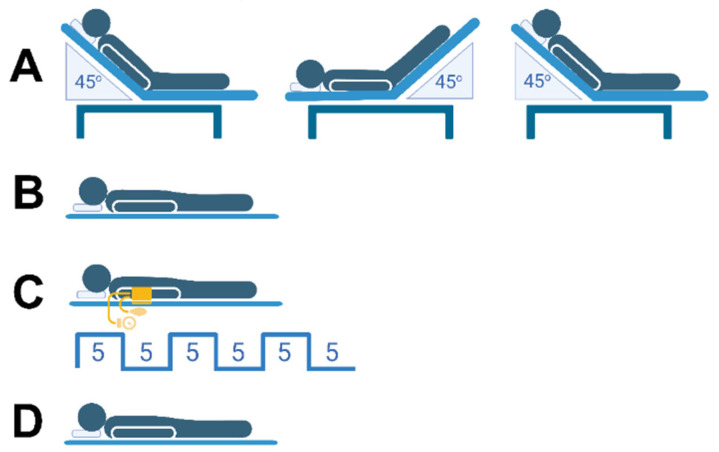
Study sequence: (**A**), passive leg raising; (**B**), rest in the supine position; (**C**), remote ischemic conditioning; (**D**), rest in the supine position. This image was created with BioRender.com.

After completing the PLR test, the participants were placed in the supine position for 5 min. Afterward, RIC was performed. The RIC protocol consisted of three cycles of upper-hand compressions using a blood pressure cuff inflated to 200 mmHg for 5 min, followed by a 5-min reperfusion period (after cuff release). During each deflation cycle, PI and HR were measured. The study sequence is illustrated in Figure 1.

Patients on mechanical ventilation received a combination of propofol and fentanyl for analgesia and sedation. Ventilator settings, vasopressor administration, and sedative drug doses were kept constant throughout the study.

### 2.3. Perfusion Index

PI was automatically calculated from the plethysmogram by the Rad-97 (Masimo corp., Irvine, CA, USA) device as the ratio of the pulsatile component to the non-pulsatile component of the light received by the pulse oximeter’s detector, expressed as a percentage. Measurements were taken using a sensor placed on the third or fourth finger, selecting the digit that showed the highest PI value on the arm fitted with the blood pressure cuff.

The predefined parameters—PI, SVI, and HR—from monitors such as the Rad-97, PiCCO_2_, or ICON were extracted in real time using the ICM+ software (version 9, University of Cambridge, UK). Data were exported from the devices at approximately 1 sample per heartbeat (~1 Hz). The extracted PI data were filtered with a third-order Butterworth low-pass filter at 0.083 Hz to match the 12-s averaging. We averaged the PI values over 12 s, the exact duration used by the PiCCO2 device for averaging pulse contour analysis-derived values. During the cuff deflation phase in RIC, the peak PI, the time to reach the peak PI, and the steady-state PI at 5 min (end of the deflation phase) were determined from the PI waveform using MATLAB software (R2021a, MathWorks, Natick, MA, USA). Maximum PI change (dPImax) was calculated as peak PI minus baseline PI.

### 2.4. Statistical Analysis

Data were analyzed using the Statistical Package for Social Sciences (IBM Corp., version 30; Armonk, NY, USA). The distribution of quantitative variables was tested with a Kolmogorov–Smirnov normality test. Since most parameters showed a non-normal distribution, data are presented as the median [interquartile range, IQR] and analyzed with non-parametric tests. Differences between groups were tested using a Mann–Whitney U test. The Friedman test was used to evaluate how parameters change over time within each group, followed by a Wilcoxon test with Bonferroni correction for multiple comparisons. Correlations were tested with a Spearman correlation test. The predictive value of PI change during the first cuff deflation on preload responsiveness was calculated using a receiver operating characteristic (ROC) curve, and the area under the curve was computed. A *p*  <  0.05 was considered statistically significant. The primary outcome of the study is the correlation between PLR-induced SVI changes and blood pressure cuff deflation-induced PI changes in critically ill patients. Based on power calculations (α = 0.05, 80% power), a sample size of approximately 33 patients is sufficient to detect moderate-to-strong correlations (r ≥ 0.5) and to test the study hypothesis that dPImax during the first cuff deflation of the blood pressure cuff in RIC is associated with changes in SVI during PLR. The secondary outcomes are differences in peak PI, time to reach peak PI, dPImax, and PI at 5 min after blood pressure cuff release during RIC between critically ill patients and healthy controls. The comparison with 14 healthy controls was intended to provide reference values. Although the control group was smaller, this sample size is sufficient to detect large effect sizes. The magnitude of the standardized mean difference effect size between groups was estimated by Cohen’s d test. In our study, the effect sizes were greater than 0.8 for all secondary outcomes that were statistically significant.

## 3. Results

### 3.1. Patient Characteristics

The baseline characteristics of the 33 patients included in the analysis are shown in Table 1. The median age was 62 years, and 67% were male. The Simplified Acute Physiology Score (SAPS) II and Sequential Organ Failure Assessment (SOFA) scores were 52 (32–60) and 8 (6–13), respectively. Fifteen patients (45%) were receiving vasopressors, and 18 (55%) were on mechanical ventilation. Fifteen patients (45%) were preload responders.

### 3.2. PI Changes After Cuff Deflation During RIC

In both critically ill patients and healthy controls, PI increased significantly following each cuff deflation (*p* < 0.005). In critical ill patients, the time to reach peak PI (time-to-peak) significantly decreased with each subsequent cycle, reaching its minimum during the third deflation (Table 2). While critically ill patients initially exhibited a significantly longer time-to-peak compared to healthy volunteers during the first and second deflations, this difference was abolished by the third cycle. Additionally, after the third cuff deflation, the 5-min PI value was not different from the baseline, and dPImax was comparable to that of healthy young individuals (Table 2).

In a subgroup analysis comparing patients with septic shock (n = 13) to those without shock (n = 18), the septic shock cohort had a significantly lower median baseline PI 2.2 (0.9–6.0%) vs. 6.0 (2.6–11.6%); *p* = 0.023). Additionally, septic shock was associated with a lower peak PI, a longer time-to-peak, and a lower PI at the five-minute mark across all three cuff deflations. While the dPImax did not differ significantly following the first and second cuff releases, it was significantly lower in the septic shock group after the third deflation (1.6 (0.6–2.3%) vs. 3.1 (1.7–6.5%); *p* = 0.041). Furthermore, patients with septic shock had significantly higher SAPS II (56 (53–65) vs. 32 (27–52); *p* = 0.001) and SOFA scores (13 (10–15) vs. 6 (6–7); *p* < 0.001) than those without shock.

### 3.3. Prediction of Preload Responsiveness

**Figure 2:**
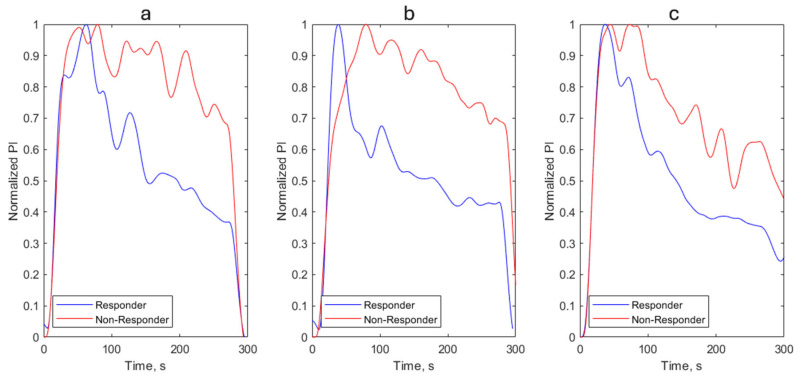
Examples of observed waveforms of the perfusion index in preload responders (blue) and nonresponders (red) after cuff deflation during remote ischemic conditioning in critically ill patients. The data are normalized to the maximum amplitude for shape comparison. (**a**) first cuff release; (**b**) second cuff release; (**c**) third cuff release.

Following the first cuff deflation, dPImax significantly correlated with SVI changes during PLR (r = 0.63, *p* < 0.001). Similarly, though weaker, correlation was observed between the 5-min post-deflation PI and SVI changes (r = 0.48, *p* = 0.007). After the first cuff deflation, if the PI absolute increased by ≥2.8%, a positive SVI response (≥10%) during PLR was observed, with a sensitivity of 64% and a specificity of 94% (area under the receiver operating characteristic curve 0.752; 95% CI, 0.564–0.940, *p* = 0.008). Examples of PI waves in preload responders and non-responders after cuff deflation during RIC are shown in Figure 2.

## 4. Discussion

This study is the first to investigate PI dynamics following brachial cuff deflation during RIC in critically ill patients. We explore these changes through two distinct clinical lenses: (1) as a novel surrogate for preload responsiveness, based on its correlation with changes in SVI during PLR, and (2) as a real-time assessment of vascular reactivity during RIC.

Our results demonstrate that the maximum PI change following brachial blood pressure cuff release after five minutes of inflation correlates with PLR-induced changes in SVI (r = 0.63). Additionally, we found that the PI response following cuff deflation may serve as a reliable surrogate for assessing preload responsiveness (AUC = 0.752). The PLR test is a well-validated method for assessing fluid responsiveness and is widely used in ICUs [16,17]. However, the PLR test is not suitable for various clinical situations, including surgery, pelvic or lower extremity fractures, and spinal cord injuries. Consequently, alternative approaches are needed to assess fluid responsiveness while avoiding these limitations. PI has been used in fluid responsiveness testing as a surrogate for cardiac output or stroke volume during various maneuvers. For example, an increase of 9% in PI during a passive leg-raising test [14], a 2.5% increase during an end-expiratory occlusion test [18], and a 7% decrease during a tidal volume challenge [19] have all been shown to predict fluid responsiveness with fair accuracy. This application represents an important advance for guiding fluid therapy in settings where cardiac output monitoring is unavailable. In contrast to the systemic effects induced by these maneuvers, our approach relied on local blood flow occlusion and reperfusion using a blood pressure cuff. This may explain why the change in PI observed during cuff release was more pronounced. Blood pressure cuff release triggers nitric oxide (NO) release during reperfusion, as the restored blood flow creates shear stress, which stimulates endothelial nitric oxide synthase to produce NO. Similarly, during PLR, increased blood flow caused by blood shifting from the legs triggers an endothelial response to shear stress. Kamran and colleagues [20] demonstrated that brachial artery dilation (BAD) occurs in response to PLR and is proportional to flow-mediated dilation (FMD) induced by brachial cuff release immediately after 5-min inflation. The magnitude of PLR-BAD was less than half that of FMD, and it appears to occur through the same endothelial-dependent mechanism as FMD. However, in clinical practice, quicker and simpler methods than brachial artery ultrasound are needed to assess vascular (endothelial) function or preload responsiveness. Zahedi and colleagues [10] demonstrated that photoplethysmographic waveform analysis can detect FMD response during brachial cuff deflation after a 4-min inflation in the peripheral circulation, producing results similar to those from ultrasound measurements of brachial artery diameter. The response time of the peak photoplethysmographic-AC wave change was significantly shorter than that of the peak brachial artery diameter measured by ultrasound. These findings support the validity of our use of PI, derived from the plethysmographic waveform, as a measure of vascular (endothelial) function during RIC.

Additionally, during RIC, we observed improved vascular reactivity, evidenced by no difference in time to reach maximal PI or the maximal PI difference after the third cuff release compared with young healthy individuals. Studies showed that short-term RIC was associated with improvements in FMD compared with sham [21]. Thus, most of those studies used four or five cycles of five minutes of cuff inflation followed by five minutes of reperfusion. In our study, we employed PI measurement instead of brachial ultrasound, and RIC involved three cycles of 5 min of cuff inflation followed by 5 min of reperfusion.

Several limitations of our study should be acknowledged. First, this was a single-center investigation with a relatively small sample size, which may constrain the generalizability of our conclusions. Although the sample size was calculated to ensure sufficient statistical power for detecting correlations, larger, multicenter studies are needed to confirm these findings across more homogeneous ICU populations. Second, the inclusion of a clinically heterogeneous ICU population limits the strength of definitive clinical inferences. However, because this was an exploratory study, such heterogeneity reflects routine clinical practice and may enhance the external validity of the findings. Third, we used a PLR maneuver to assess preload responsiveness. Despite possible limitations, this method remains the most extensively validated and widely used across different patient populations cohorts [17,22]. Fourth, our analysis focused exclusively on PI measured at the finger, even though its relationship with stroke volume may vary depending on the anatomical site of measurement. Fifth, we averaged the real-time PI values over 12 s, as this was the only feasible method for aligning them with pulse contour analysis–derived values, which are averaged over a 12-s interval [14]. Sixth, clinical observational studies show poor agreement between cardiac output estimates from transthoracic thermodilution and those from electrical cardiometry in critically ill patients [23,24]. Therefore, we did not compare cardiac output or stroke volume between critically ill patients and healthy volunteers.

## 5. Conclusions

The maximum change in perfusion index following brachial blood pressure cuff release after five minutes of inflation serves as a noninvasive surrogate for stroke volume changes during PLR and may help identify preload responsiveness in critically ill patients. Furthermore, the equalization of PI kinetics between critically ill patients and healthy controls by the third RIC cycle suggests that RIC may acutely improve vascular reactivity in the critically ill. However, additional studies are needed to verify a direct relationship between PI changes and endothelial function during RIC, to determine whether the effect is systemic, and to characterize its duration in critically ill patients.

## Figures and Tables

**Table 1:** Characteristics of the study subjects.

	Critical Ill Patients	Healthy Subjects
Age, years	62 (49–74)	27 (24–30)
Male sex, *n* (%)	22 (67)	8 (57)
Body mass index	27.6 (24.2–30.2)	24.2 (22.1–27.2)
SAPS II score	52 (32–60)	-
SOFA score at inclusion	8 (6–13)	-
Comorbidities, n (%)		-
Hypertension	21 (64)	
Diabetes	5 (15)	
Chronic kidney disease	5 (15)	
Cancer	9 (27)	
After liver transplantation	7 (21)	
Type of shock, n (%)		-
Septic	13 (39)	
Hypovolemic	1 (3)	
Distributive nonseptic	1 (3)	
Dose of Norepinephrine, n, median (interquartile range), µg/kg/min	15, 0.10 (0.04–0.16)	
Mechanical ventilation, n (%)	18 (55)	-
Baseline HR (before RIC), beats/min	84 (76–99)	69 (58–74)
Baseline PI (before RIC), %	4.5 (1.8–9.0)	6.5 (1.6–9.4)

Data are presented as the median (25th–75th) or number (n) and frequency (%). SAPS II, Simplified Acute Physiology Score; SOFA, Sequential Organ Failure Assessment; HR, heart rate; PI, perfusion index; RIC, remote ischemic conditioning.

**Table 2:** Perfusion index changes after blood cuff deflation in remote ischemic conditioning.

	Def1	Def2	Def3	*p*
Peak PI, %				
Critical ill patients	6.7 (5.3–11.3) *	7.0 (4.2–11.0) *	7.0 (4.4–11.9) *	0.152
Healthy	14.1 (5.0–17.3) *	11.6 (4.7–17.8) *	12.0 (4.9–17.4) *	0.558
Time to reach peak PI, sec				
Critical ill patients	85.7 (55.6–96.7) ^x^	69.0 (46.9–94.1) ^ax^	67.8 (36.9–90.7) ^ab^	0.005
Healthy	59.0 (31.0–63.7)	51.2 (31.3–70.9)	47.0 (31.3–65.2)	0.264
PI at 5 min, %				
Critical ill patients	6.0 (2.2–9.8) *	5.5 (3.8–8.7) *	4.9 (2.0–8.4)	0.177
Healthy	8.9 (3.0–17.5)	7.2 (2.3–11.0)	4.0 (2.5–14.2)	0.150
dPImax, %				
Critical ill patients	1.5 (1.0–3.5) ^x^	1.7 (0.6–4.6) ^x^	2.1 (0.4–3.4)	0.267
Healthy	4.9 (2.2–9.3)	3.4 (1.9–8.7)	3.8 (2.1–8.1)	0.307

Data are presented as the median (25th–75th). PI, perfusion index; dPImax, the difference between Peak PI and baseline PI; Def1, first cuff deflation after first inflation; Def2, second cuff deflation after second inflation; Def3, third cuff deflation after third inflation. ^a^ *p* < 0.02 vs. Def1; ^b^ *p* < 0.001 vs. Def2; ^x^ *p* < 0.05 comparing critical ill patients with healthy subjects. * *p* < 0.05 compared to the baseline value.

## Data Availability

The datasets generated in this study are available from the corresponding author upon request.

## Abbreviations

The following abbreviations are used in this manuscript:

RIC	Remote ischemic conditioning
PI	Perfusion index
FMD	Flow-mediated dilation
PLR	Passive leg raising
PiCCO	Pulse indicator continuous cardiac output
SVI	Stroke volume index
HR	Heart rate
SAPS II	Simplified Acute Physiology Score
SOFA	Sequential Organ Failure Assessment
NO	Nitric oxide
BAD	Brachial artery dilation
